# Stanniocalcin‐2 inhibits skeletal muscle growth and is upregulated in functional overload‐induced hypertrophy

**DOI:** 10.14814/phy2.15793

**Published:** 2023-08-11

**Authors:** Arimantas Lionikas, Ana I. Hernandez Cordero, Audrius Kilikevicius, Andrew M. Carroll, Guy S. Bewick, Lutz Bunger, Aivaras Ratkevicius, Lora K. Heisler, Mette Harboe, Claus Oxvig

**Affiliations:** ^1^ School of Medicine, Medical Sciences and Nutrition University of Aberdeen Aberdeen UK; ^2^ Centre for Heart Lung Innovation University of British Columbia, St. Paul's Hospital Vancouver Canada; ^3^ Department of Health Promotion and Rehabilitation Lithuanian Sports University Kaunas Lithuania; ^4^ The New Zealand Institute for Plant & Food Research Limited Palmerston North New Zealand; ^5^ Animal Genetics Company (AnGeCo) Edinburgh Scotland; ^6^ Department of Molecular Biology and Genetics Aarhus University Aarhus Denmark

**Keywords:** IGF, resistance training, skeletal muscle, STC2

## Abstract

**Aims:**

Stanniocalcin‐2 (STC2) has recently been implicated in human muscle mass variability by genetic analysis. Biochemically, STC2 inhibits the proteolytic activity of the metalloproteinase PAPP‐A, which promotes muscle growth by upregulating the insulin‐like growth factor (IGF) axis. The aim was to examine if STC2 affects skeletal muscle mass and to assess how the IGF axis mediates muscle hypertrophy induced by functional overload.

**Methods:**

We compared muscle mass and muscle fiber morphology between *Stc2* ^*−/−*^ (*n* = 21) and wild‐type (*n* = 15) mice. We then quantified IGF1, IGF2, IGF binding proteins −4 and −5 (IGFBP‐4, IGFBP‐5), PAPP‐A and STC2 in plantaris muscles of wild‐type mice subjected to 4‐week unilateral overload (*n* = 14).

**Results:**

*Stc2* ^*−/−*^ mice showed up to 10% larger muscle mass compared with wild‐type mice. This increase was mediated by greater cross‐sectional area of muscle fibers. Overload increased plantaris mass and components of the IGF axis, including quantities of IGF1 (by 2.41‐fold, *p* = 0.0117), IGF2 (1.70‐fold, *p* = 0.0461), IGFBP‐4 (1.48‐fold, *p* = 0.0268), PAPP‐A (1.30‐fold, *p* = 0.0154) and STC2 (1.28‐fold, *p* = 0.019).

**Conclusion:**

Here we provide evidence that *STC2* is an inhibitor of muscle growth upregulated, along with other components of the IGF axis, during overload‐induced muscle hypertrophy.

## 1 INTRODUCTION

Skeletal muscle mass can differ over twofold between the same sex, height, and age individuals (Hernandez Cordero et al., 2019) impacting the susceptibility to sarcopenia, the aging‐related loss of muscle mass and function. Genetic variability accounts for 36%–80% of muscle mass differences in humans (Cruz‐Jentoft et al., 2010; Hernandez Cordero et al., 2019; Livshits et al., 2016), and conceivably influences cellular mechanisms underlying development, growth, and maintenance of tissue. However, specific genes contributing to this heritability remain elusive.

Sustained resistance exercise training can also induce an increase in muscle mass through hypertrophy of muscle fibers (MacDougall et al., 1984), which in part is mediated by insulin‐like growth factor‐1 (IGF1). For instance, in rodent models, exogenous delivery of IGF1 (Adams & McCue, 1985), or its overexpression in muscle tissue (Oliver et al., 2005), results in hypertrophy of the treated muscles. Additionally, overload protocols which simulate resistance exercise training in rats, trigger an increase in the concentration of IGF1 (Adams et al., 1985). Indeed, a single session of resistance exercise is sufficient to upregulate expression of IGF1 in the exercised muscle in humans (Bamman et al., 2001). These findings strongly suggest that the IGF axis is mediating the upstream response to exercise in skeletal muscle, but it could also mediate the effects of genetic variability.

The IGF1 binds to IGF1 receptors on the sarcolemma and triggers intracellular activation of the PI3K‐Akt/PKB‐mTOR cascade (Rommel et al., 2001) leading to upregulation of muscle protein synthesis (Butterfield et al., 1997). While IGF1 is synthesized and released by muscle fibers, its autocrine and/or paracrine interaction with its receptor is modified by competitive binding to an extracellular pool of IGF binding proteins (IGFBPs) (Oxvig & Conover, 2023). Two of the six IGFBP family members, IGFBP‐4 and IGFBP‐5, which are predominantly transcribed, synthesized and secreted by muscle fibers, constitute a potent regulatory level of free interstitial IGF1. For instance, overexpression of IGFBP‐5 markedly retards skeletal muscle growth in mice implicating a growth suppressing role (Salih et al., 2004). IGFBP‐4 on the other hand promotes growth, as its deletion in vivo causes a growth deficit (Ning et al., 2008). This is because IGFBP‐4 helps deliver the IGF ligand to the proximity of its receptor on the cell surface, thereby facilitating the ligand‐receptor interaction following IGFBP‐4 cleavage (Oxvig, 2015).

Expression of IGFBP‐4 and IGFBP‐5 in skeletal muscle substantially exceeds that of IGF1 (Lionikas et al., 2012). Furthermore, in serum around 99% of IGF1 is in complex with IGFBPs (Frystyk et al., 1994). Importantly, IGFBP binding does not permanently neutralize IGF1, but instead it establishes an interstitial reservoir of IGF:IGFBP complex from which IGF1 can be mobilized by proteolytic cleavage of IGFBPs. Pregnancy associated plasma protein A (PAPP‐A) (Conover & Oxvig, 2023) specifically cleaves IGFBP‐4 (Lawrence et al., 1999) and IGFBP‐5 (Laursen et al., 2001), both with high catalytic efficiency (Gyrup et al., 2007) and is also highly expressed in skeletal muscle (Harstad & Conover, 2014). Furthermore, studies have shown that PAPP‐A overexpression in skeletal muscle increases free IGF1 and results in muscle hypertrophy (Deb et al., 2012; Rehage et al., 2007). Hence, there is strong evidence that PAPP‐A upregulates anabolic processes by increasing free IGF1 in the interstitium surrounding muscle fibers.

Recently, an additional level of IGF axis regulation has been described. Stanniocalcin‐2, encoded by the *STC2* gene, binds PAPP‐A and thereby inhibits its proteolytic activity in vitro (Jepsen et al., 2015 *;* Kobberø et al., 2022). In vivo, overexpression of STC2 stunts somatic growth and reduces skeletal muscle mass by 15%–32% (Gagliardi et al., 2005), while conversely, its deletion leads to a 15% body weight increase although the effects on skeletal muscle was not analyzed in that model (Chang et al., 2008). Importantly, *STC2*'s involvement in regulating skeletal muscle mass has also been implicated in a human GWAS (Hernandez Cordero et al., 2019). A rare variant of *STC2*, which showed reduced PAPP‐A inhibiting capacity (Marouli et al., 2017), is associated with increased appendicular muscle mass (Hernandez Cordero et al., 2019). Thus, although *STC2* seems a strong regulator of muscle mass, these studies do not reveal what muscle fiber parameter is affected, that is, if *STC2* influences the number of muscle fibers, their size or both. Furthermore, it remains unclear if *STC2* plays a role in muscle response to resistance training.

Altogether, several lines of evidence implicate the IGF axis in regulating muscle mass. However, it remains less clear if it plays an equally important role in adaptation to the exercise‐induced muscle hypertrophy and in muscle mass accrual resulting from the effects of genetic variability. Therefore, to improve our understanding of the role of *STC2* and other components of the IGF axis we characterized the effect of *Stc2* on muscle morphology (fiber cross‐sectional area (CSA) and fibre number) in an *Stc2* knockout mouse model. We also examined a panel of six IGF axis components, IGF1, IGF2, IGFBP‐4, IGFBP‐5, PAPP‐A, and STC2, in skeletal muscles differing in size either due to overload‐induced hypertrophy or because of the genetic background.

## 2 METHODS

### 2.1 Stc2 knockout study

*Stc2* ^*−*/*−*^ mice were generated using CRISPR‐Cas9 genome editing. Eight guide RNAs (gRNAs) targeting STC2 exon 1 were tested in ES cells and their efficiency and frequency of indels introducing frameshift was determined by TIDE analysis (Brinkman et al., 2014). One gRNA was chosen for targeting of the gene (gRNA sequence: 5′‐ GACTCCACGAACCCTCCGGA‐3′). Plasmids expressing this gRNA and Cas9 were used for pronuclear injection into fertilized C57BL/6J eggs, which were then transferred to foster mice. Progeny were genotyped by Sanger sequencing after standard PCR amplification of DNA from ear biopsies (Primers: Fw: 5′‐CTGGGTAACCTCTATCCGAGC‐3′, Rv: 5′‐CGGGGAAGGCTAGCAACAAG‐3′). The mutation deleted 22 nucleotides in exon 1 (CGGACTCCACGAACCCTCCGGA, nt. 273–294 of NCBI NM_011491.3) causing a frameshift and thus introduced a premature stop codon in exon 2. Mice were housed at Aarhus University and kept on a 12 h light/12 h dark cycle with access to food pellets (Altromin 1324) and water ad libitum. All mouse work was conducted with the permission of the Danish Animals Ethics Council (permit number 2017‐15‐0202‐00055).

*Stc2* ^*−*/*−*^ (14 females and 7 males) and wild‐type C57BL/6 (7 females and 8 males) mice were sacrificed at 90 ± 1 days of age. The carcasses were transferred to ultralow freezer (−80°C) and shipped on dry ice to Dr Lionikas laboratory at the University of Aberdeen.

On the day of dissection, the carcases were thawed and two dorsiflexors, tibialis anterior (TA) and extensor digitorum longus (EDL), and three plantar flexors, gastrocnemius (gastroc), plantaris and soleus, were bilaterally removed under a dissection microscope and weighed to a precision of 0.1 mg on a balance (Pioneer, Ohaus). Muscles then were snap frozen in isopentane chilled by liquid nitrogen and transferred to an ultralow freezer. The tibia was detached at the knee and ankle joints and its length measured to a precision of 0.01 mm with a digital caliper (Z22855, OWIM GmbH & Co) as a proxy of the axial dimensions of the muscles. As studies revealed, Stc2^−/−^ and wild‐type samples showed a strong and positive association between the corresponding traits of the left and right hindlimb, with a Pearson correlation of homologous muscle weight ranged between 0.81 and 0.99, whereas tibial length correlation (left–right) was 0.89. Data of only the left limb are presented.

The EDL showed the most robust difference in weight and therefore selected for histological analyses. The left EDL muscle of *Stc2* ^*−/−*^ and wild‐type males were cut transversely in 7 μm thick sections with a cryotome (Leica CM1850UV) at −23°C and collected on a positively charged glass slide (Superfrost Plus, Menzel Gläser). Sections were air dried and stored at −70°C. On the day of staining, the sections were blocked in 10% foetal calf serum in PBS blocking buffer, followed by a 2‐h incubation in blocking buffer containing cocktail antibodies against Type IIa myosin 1:200 (SC‐71, DSHB) and laminin 1:50 (L9393, Sigma Aldrich). Following three 5‐min washes in PBS, a cocktail of secondary antibodies (Alexa goat anti‐mouse 488 IgG1, 1:500, and Alexa goat anti‐rabbit 594, 1:200) in blocking buffer were applied for 1 h to visualize location of myosin and laminin, respectively. After incubation, the slides were washed in PBS (three times for 5 min), blotted, and Mowiol 4–88 (Sigma‐Aldrich) was added over the sections and covered with a coverslip sealing it afterwards with nail varnish.

Microscopy was performed in the Microscopy and Histology Core Facility at the University of Aberdeen. Slides were scanned using Axioscan Z1 slide scanner (Zeiss) with 10× magnification objective. The images were analyzed using a semi‐automatic process in ImageJ software (Schneider et al., 2012) for the number and CSA of muscle fibers. After setting the scale and splitting color channels, the red and green channel images were converted to binary using the thresholding function. To determine the CSA of the fibers and fiber number, the red channel image (capturing laminin) was processed using the binary functions *Dilate* and *Erode* to enhance the boundaries of all muscle fibers. It was followed by the *Analyze Particles* function to obtain area and count measured fibers with a setting of 0.4–1.0 for circularity to exclude erroneous objects within the expected size range, 50–4500 μm^2^, but not consistent with muscle fiber shape. The analysis output was visually inspected and, in the instances where adjacent fibers appeared connected/incompletely separate by the process, the connections were manually deleted before repeating the analysis. The number and CSA of Type IIA fibers was determined using the *Analyze Particles* function in the green channel (capturing Type IIA myosin) image.

### 2.2 Chronic overload study

To reduce usage of animals, samples for these analyses were obtained from a previous collaborative study carried out in Lithuania (Kilikevicius et al., 2016). All animal procedures were approved by the Lithuanian State Food and Veterinary Service (Ref. #0230). Briefly, the BALB/cByJ (*n* = 4 male), C57BL/6J (*n* = 4 male and *n* = 2 female) and DBA/2 J (*n* = 2 male and *n* = 2 female) were subjected to unilateral ablation of the gastrocnemius muscle and otherwise identical sham surgery on the contralateral leg at the age of 90 ± 3 days of age. The animals were sacrificed 4 weeks later, and the plantaris muscle excised bilaterally, weighed, snap frozen and kept in an ultralow freezer. The effect of overload was stereotypical regardless of the grouping variables. Because of that, due to limited sample size across strains and unbalanced male to female ratio, no strain or sex effects were examined.

### 2.3 BEH and BEH^C^ ^/+^ mouse strain study

This experimental model was chosen because the relative difference in the weight of homologous muscles between the BEH and BEH^*C*/+^ mice was comparable to the hypertrophy observed in the chronic overload study. Muscle samples for these analyses were obtained from a previously reported study (Lionikas et al., 2013). The BEH strain was developed from a heterogeneous base population of mice in Berlin, Germany by divergent selection for high body weight (the BEL strain was developed in the same study by selection for low body weight) (Bunger et al., 2001). It has been later discovered that homozygosity of a mutant myostatin (*Mstn*) gene variant, known as the *Compact* allele is partially responsible for a hypermuscular phenotype of BEH strain (Szabo et al., 1998). The BEH^*C*/*+*^ mice in the present study, heterozygous for the *Compact* allele, are a progeny of a cross between the BEH and BEH^+/+^ strain mice. The BEH^+/+^ mouse strain was created by crossing the BEH and BEL strains and then repeatedly backcrossing the offspring to BEH for at least 17 generations using marker assisted selection for the wild‐type myostatin (Amthor et al., 2007). Hence the BEH and BEH^*C*/+^ mice provide a model of largely monogenic effect on skeletal muscle mass. Importantly, muscle mass between BEH and BEH^*C*/+^ mice primarily differ due to the CSA of muscle fibers and the proportion of the glycolytic fibers but not the total number of fibers (Lionikas et al., 2013). These mouse lines were developed at the University of Edinburgh as described earlier (Amthor et al., 2007). The experimental animals used in this study were maintained there as well and euthanized under schedule 1 of UK Home Office regulations. Plantaris muscle samples of BEH (*n* = 5) and BEH^*C*/+^ (n = 5) male mice were used in the analyses.

### 2.4 Enzyme‐linked immunosorbent assay

Frozen samples of plantaris muscles were crushed with a plastic pestle in 1.5 mL tube on dry ice, then homogenized in 10 μL of PBS (supplemented with protease inhibitor cocktail; Sigma‐Aldrich) per milligram of tissue on crushed ice for at least 2 min. Samples were subjected to three freeze (−20°C)—thaw cycles, spun at 10,000 g for 10 min at 4°C and the supernatant was then collected and stored at −20°C for later analyses.

The concentration of six IGF axis components was measured using enzyme‐linked immunosorbent assay (ELISA) kits (LifeSpan Biosciences, Inc.): IGF1 (LS‐F3417), IGF2 (LS‐F5071), IGFBP‐4 (LS‐F5478), IGFBP‐5 (LS‐F36619), PAPPA (LS‐F4037), and STC2 (LS‐F57455) following manufacturer's recommendations. All dilutions for the standard curve and the majority of samples were run in duplicate (in a few cases duplicate analysis was not possible due to small volume). Median (and minimum; maximum) coefficient of variation was 2.9% (0%; 24.6%) and 2.8% (0%; 38.4%) for the standard curve dilutions and samples, respectively. Dilutions of the standard in all assays fit a linear or polynomial trend with *R* ^2^ > 0.99.

### 2.5 Statistics and data analysis

The strength of the bilateral association between corresponding traits was examined using Pearson correlation. Muscle weight in C57BL/6 wild‐type and *Stc2* ^*−*/*−*^ mice was analyzed using a linear model, weight ~ sex + genotype. For the analyses of muscle mass and protein level, a *t*‐test was used to compare between wild‐type and knockout, BEH and BEH^*C*/+^, or a paired *t*‐test for control and overloaded muscle samples comparison. The Kolmogorov–Smirnov test indicated significant deviation of the fiber CSA from a normal distribution therefore medians were used for comparison between the *Stc2* ^*−*/*−*^ and wild‐type mice. To compare the distribution of fiber size, all fibers from the *Stc2* ^*−*/*−*^ or wild‐type mice were pooled and histograms generated with 156 μm^2^ bins. A Chi‐squared test was used (followed by the Bonferroni correction to counteract the multiple comparison problem) to test a within‐bin difference in fiber count. Data are presented as mean and standard deviation unless stated otherwise.

## 3 RESULTS

### 3.1 *Stc2* knockout increased skeletal muscle mass

**FIGURE 1:**
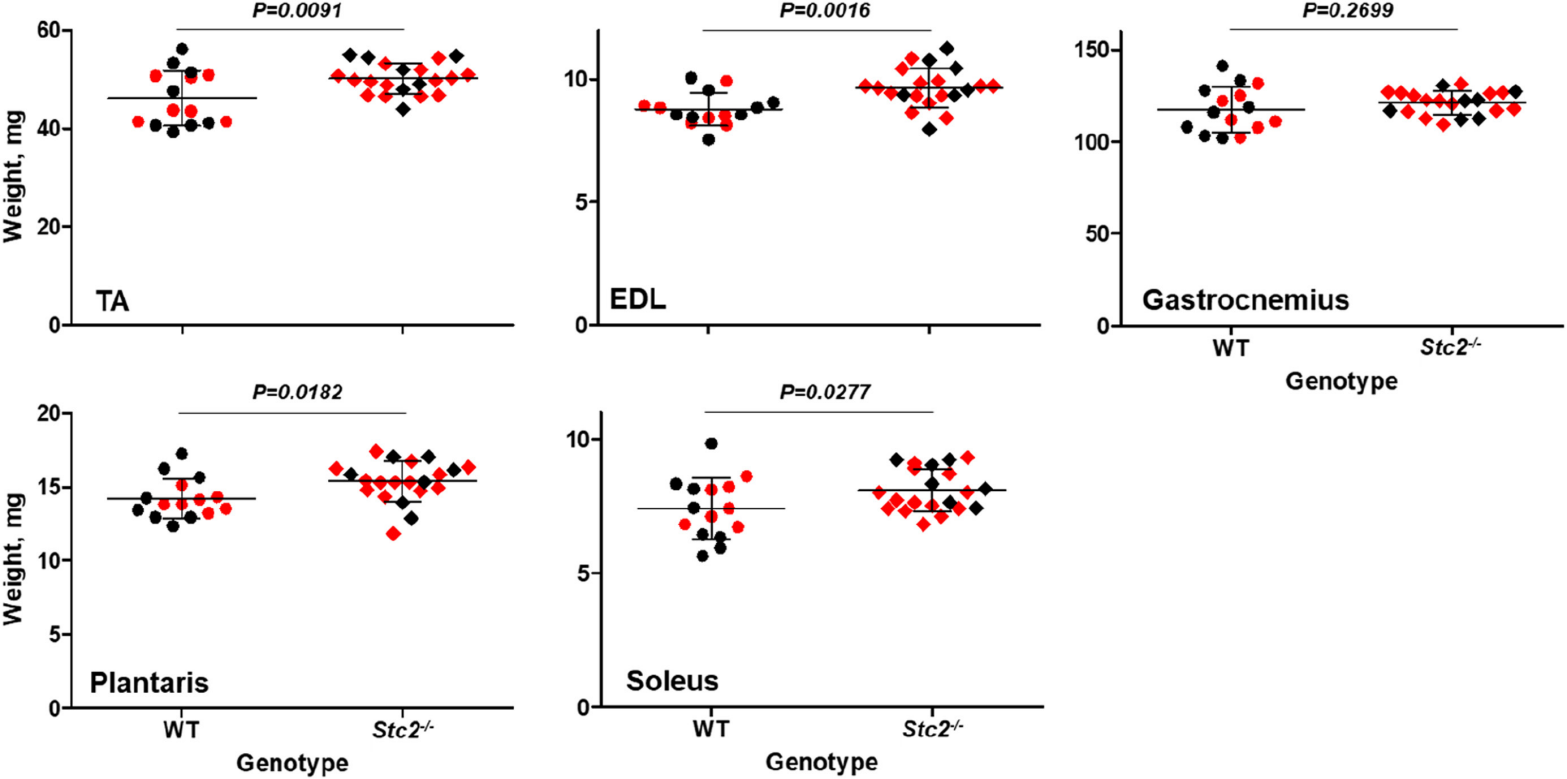
*Stc2* effects on muscle weight. Plots illustrate the relationship between weight of five hindlimb muscles (y‐axis, weight residuals adjusted for sex plus overall mean) and the genotype of mice (x‐axis, wild type [WT, *n* = 15] or knockout [*Stc2* ^*−*/*−*^, *n* = 21]). Red symbols—female, black—male. The horizontal line represents the mean value, the error bars represent standard deviation. *p*‐values of general linear model (with sex and genotype as factors) are shown for the genotype effect.

Consistent with our hypothesis that STC2 is a negative regulator of muscle mass, muscles of the *Stc2* ^*−/−*^ mice were 8%–10% larger than in the wild‐type for TA (*p =* 0.0091), EDL (*p* = 0.0016), plantaris (*p* = 0.0182) and soleus (*p* = 0.0277) although only 3% (not reaching statistical significance; *p* = 0.2699) for gastrocnemius (Figure 1). All five muscles were larger in males than females (*p* < 0.0001), but similarly affected by the *Stc2* deletion in both sexes (genotype‐by‐sex *p*‐value: TA 0.739, EDL 0.816, gastrocnemius 0.586, plantaris 0.796 and soleus 0.227). The mass of a muscle is a function of its axial length and girth; hence this difference could reflect an increase in either, or both. However, the observed effect on the *Stc2* ^*−*/*−*^ muscles was not driven by the axial dimensions because tibia length did not differ between the *Stc2* ^*−/−*^ and wild‐type mice (females, 17.4 ± 0.4 mm and 17.4 ± 0.3 mm (*p* = 0.87) respectively, or males 17.9 ± 0.3 mm and 17.6 ± 0.4 mm (*p* = 0.05), respectively). Thus, the observed muscle weight difference is primarily mediated by an increase in the muscle girth.

**FIGURE 2:**
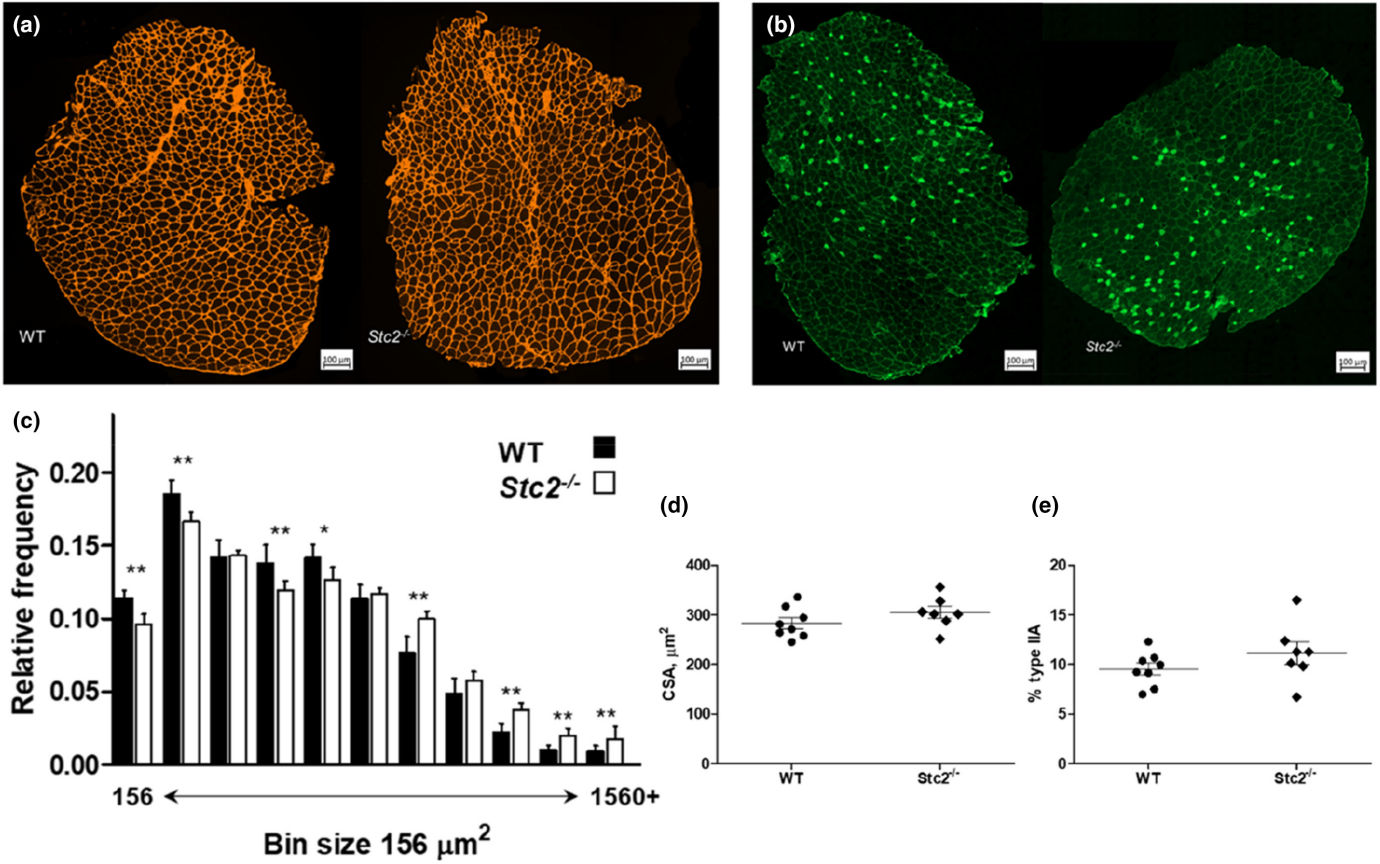
Effects of *Stc2* on muscle fibers. (a) Representative micrographs of wild‐type (WT) and *Stc2* ^*−*/*−*^ EDL muscles stained with antibodies against laminin. (b) Representative micrographs of EDL muscles stained with antibodies against type IIa myosin. (c) Relative frequency of 16,366 EDL muscle fibers from wild‐type (*n* = 8) and *Stc2* ^*−*/*−*^ (*n* = 7) males by size. Chi‐squared p‐values of the within‐bin actual versus expected number of fibers comparison were adjusted for the number of bins; *p < 0.05, **p < 0.01. (d) Cross‐sectional area (CSA) of Type IIA fibers and (e) proportion of Type IIA fibers in EDL muscle. Mean and SEM.

Muscle girth in turn depends on two factors: the number and CSA of encompassed muscle fibers. To determine if the observed muscle hypertrophy was mediated by an increase in the number of fibers, their CSA or both, we studied muscle fiber morphology histologically in sections of male EDL stained for laminin and Type IIA myosin (Figure 2a,b). The EDL muscle was selected because of the most prominent effect of *Stc2* deletion. In total 16,366 fibers were analyzed across 15 muscle samples. The number of fibers per muscle was very similar in the *Stc2* ^*−*/*−*^ and wild‐type EDL, being 1107 ± 47 versus 1073 ± 50 (*p* = 0.21), respectively. Similarly, the type IIA fiber proportions comprised 10 ± 2% of *Stc2* ^*−*/*−*^ EDL and 11 ± 3% in wild‐type EDL (Figure 2e), so neither did their number differ between the two genotypes (*p* = 0.21). This suggested that the primary effect of *Stc2* deletion is on CSA of muscle fibers.

Median CSA of all EDL muscle fibers was 8% larger in the *Stc2* ^*−*/*−*^ compared with the wild‐type mice, 591 ± 54 μm^2^ and 548 ± 65 μm^2^, respectively, although the difference did not reach statistical significance (*p* = 0.18). The distribution of the CSA revealed that the number of smaller fibers per muscle (i.e., fibers of ~700 μm^2^ or less) is higher in wild‐type EDL, whereas the number of larger fibers (i.e., fibers of ~1000 μm^2^ or more) is over‐represented in the *Stc2* ^*−*/*−*^ samples (Figure 2c). Analysis specifically of Type IIA fibers showed they tended to be larger in the *Stc2* ^*−*/*−*^ than wild‐type muscles (305 ± 32 μm^2^ and 283 ± 31 μm^2^, respectively; Figure 2d) although the difference did not reach statistical significance (*p* = 0.21). Collectively, therefore, these data show that the effect of *Stc2* on skeletal muscle mass is primarily mediated by regulating fiber size.

### 3.2 IGF axis mediates the effects of functional overload in skeletal muscle

**FIGURE 3:**
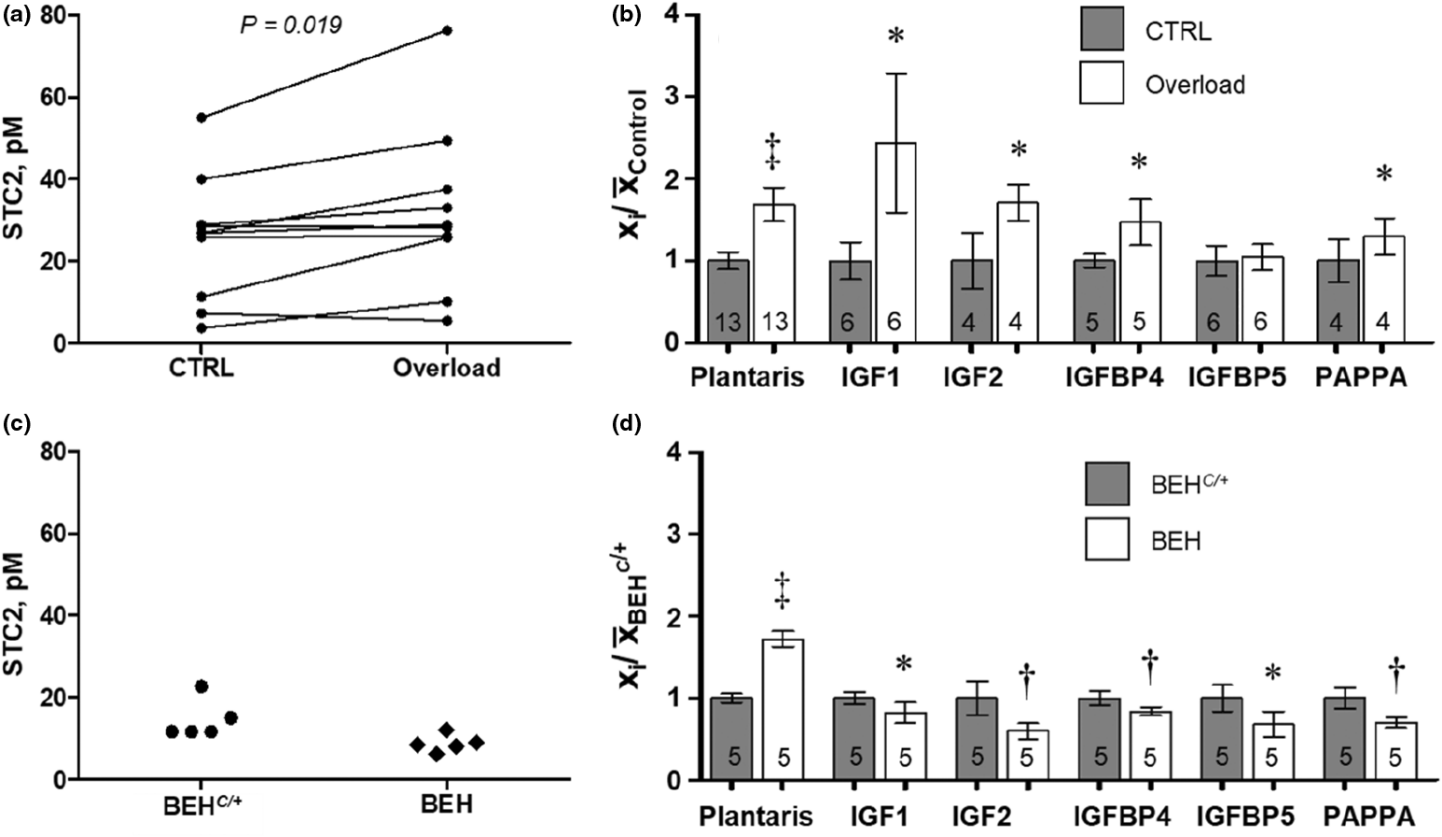
The IGF axis components in the overload‐induced muscle hypertrophy and myostatin deficiency model. (a) Concentration of STC2 following unilateral 4‐week functional overload. (b) Plantaris weight and concentration of the ligands and regulatory components of the IGF axis following 4‐week functional overload. (c) STC2 concentration in myostatin deficient BEH mice and mice heterozygous for wildtype myostatin BEH^*C*/+^. (d) Plantaris weight and concentration of the ligands and regulatory components of the IGF axis in myostatin deficient BEH mice and mice heterozygous for wildtype myostatin BEH^*C*/+^. Data are presented as mean and SD adjusted to the mean of the Control (x̄_Control_) or BEH^*C*/*+*^ (x̄_BEH_^*C*/+^) group of the corresponding variable. *p < 0.05, ^†^ *p* < 0.01, ^‡^ *p* < 0.0001 compared with the corresponding Control and BEH^*C*/+^ in (b) and (d), respectively. A paired *t*‐test was carried out in (a) and (b) and unpaired t‐test in (c) and (d). Numbers in (b) and (d) indicate sample size.

To examine the role of *Stc2* in the context of other components of the IGF axis, we mimicked resistance exercise training in laboratory mice by ablating the gastrocnemius muscle, leaving the remaining synergist plantaris overloaded. A 4‐week overload of plantaris in this manner led to a ~1.7‐fold hypertrophy of the muscle; the mean plantaris weight in the sham operated leg was 11.9 ± 1.4 mg whereas the overloaded plantaris was 20.1 ± 2.8 mg (*p =* 1.91 *×* 10^−9^). The concentration of STC2, albeit varied among the samples (Figure 3a), increased in response to the overload from 25 ± 15 pM to 32 ± 20 pM (*p* = 0.019). When the other components of the IGF axis were quantified, the hypertrophied muscles showed a significantly higher concentration of IGF1 (94 ± 33 pM vs. 39 ± 9 pM; *p* = 0.0117), IGF2 (167 ± 21 pM vs. 98 ± 34 pM, *p* = 0.0461), IGFBP‐4 (80 ± 15 pM vs. 54 ± 4 pM; *p* = 0.0268), and PAPP‐A (35 ± 6 pM vs. 27 ± 7 pM; *p* = 0.0154). Only IGFBP‐5 was not altered (787 ± 120 vs. 754 ± 136 pM; *p* = 0.6496), compared to the control muscles (Figure 3b). Thus, apart from IGFBP‐5, all of the tested IGF axis components in skeletal muscle were upregulated by the overload treatment.

### 3.3 The IGF axis is not upregulated in larger muscles of myostatin deficient mice

To test further the importance of STC2 and other proteins of the IGF axis in regulating muscle mass, we examined a hypermuscular model driven by a mutant variant of myostatin, a potent regulator of muscle mass. The genetic difference between the BEH^*C*/+^ mice and the BEH strain, is restricted to a small (~0.5 Mb) region on Chromosome 1 which harbors the myostatin encoding gene, *Mstn*, an established suppressor of muscle growth (McPherron et al., 1997). The BEH strain is homozygous for the *Compact* allele of *Mstn* conferring a hypermuscular phenotype (Varga et al., 2003), whereas BEH^*C*/+^ mice are heterozygous, carrying one wild‐type allele (see Methods). Consistent with the established role of *Mstn*, BEH plantaris muscle was ~1.8‐fold larger than in BEH^*C*/+^, 40.2 ± 2.2 mg and 23.4 ± 1.3 mg (*p* = 2.41 *×* 10^−4^; Figure 3d). However, in contrast to the overload model, a decrease, although not reaching statistical significance (*p* = 0.1008), was observed for STC2 in BEH compared to BEH^*C*/+^ mice, 16 ± 5 pM vs. 22 ± 6 pM, respectively (Figure 3c). And when the other component of the IGF axis were analyzed, they actually had a significantly lower concentration in BEH than BEH^*C*/+^ mice; IGF1 (42 ± 6 pM vs. 51 ± 4 pM, respectively; *p* = 0.0283), IGF2 (88 ± 14 pM vs. 148 ± 30 pM; *p* = 0.0041), IGFBP‐4 (81 ± 5 pM vs. 97 ± 8 pM; *p* = 0.0067), IGFBP‐5 (993 ± 227 pM vs. 1457 ± 237 pM; *p* = 0.0134) and PAPP‐A (47 ± 4 pM vs. 67 ± 9 pM; *p* = 0.0017; Figure 3d). Thus, most of the tested IGF axis components in skeletal muscle were significantly lower despite of ~1.8‐fold larger muscle mass in this model.

## 4 DISCUSSION

The main findings of this study were, (1) that *Stc2* ablation in mice increases muscle mass by up to 10% and that this is brought on by hypertrophy of the muscle fibers, rather than an increase in fiber number; (2) that functional overload‐induced muscle hypertrophy is associated with upregulation of the majority of the examined IGF axis components; (3) however, a substantially larger muscle mass in the myostatin deficient BEH mice is maintained without an upregulation of the IGF axis.

**FIGURE 4:**
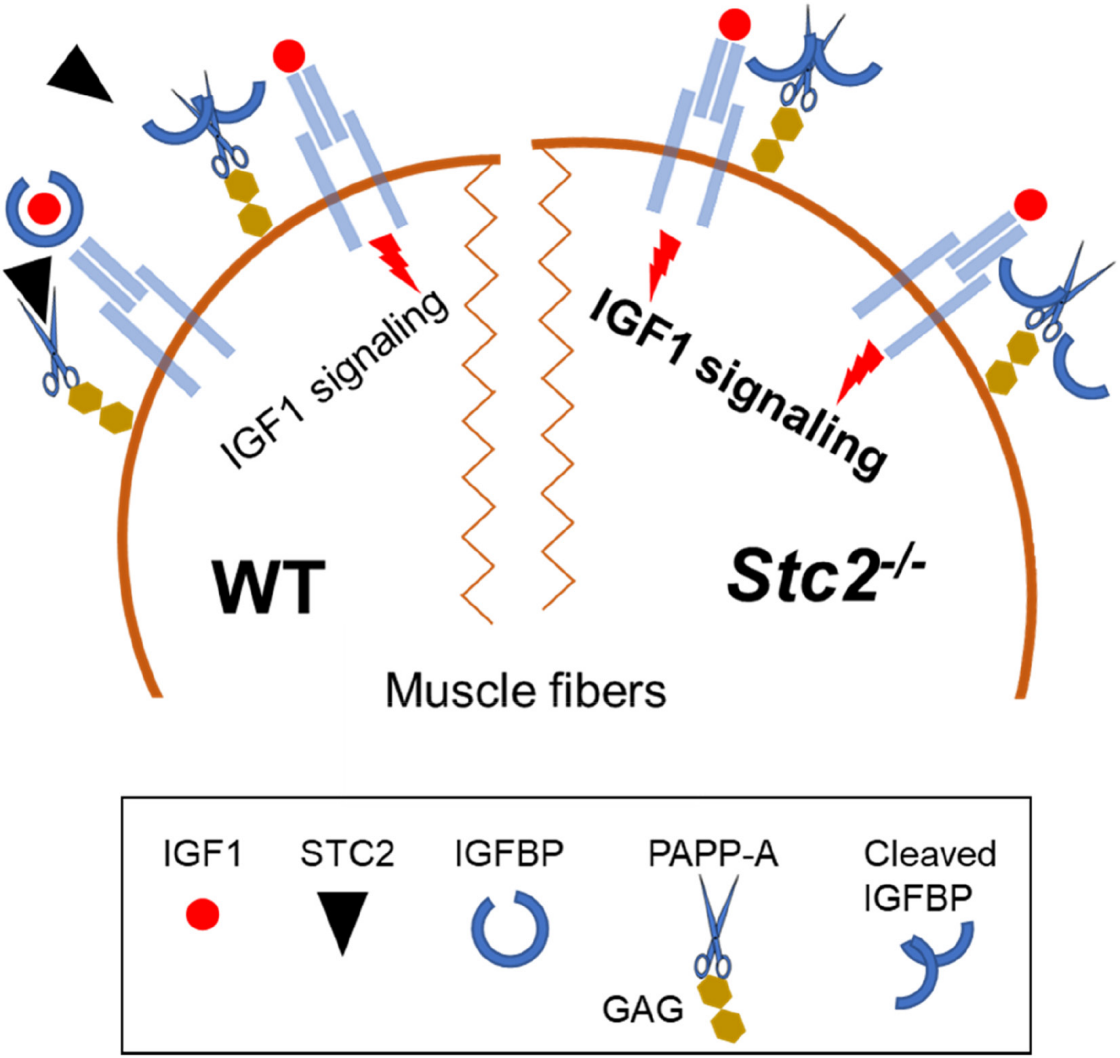
The role of STC2 in IGF axis mediated growth signaling in skeletal muscle. Intracellular signaling is triggered by the IGF1 binding to its receptor, IGF1R, and leads to upregulation of protein synthesis and growth. However, the pericellular availability of biologically active IGF1 is tightly regulated. The IGF binding proteins (IGFBP) sequester the IGF1 into the IGF1:IGFBP complex. The complex cannot interact with the receptor, but it creates a reservoir from which IGF1 can be mobilized by a specialized proteinase, PAPP‐A. PAPP‐A is tethered to the sarcolemma via glycosaminoglycans (GAG), therefore upon IGFBP cleavage biologically active IGF1 is released in close proximity to the receptor. In wildtype muscle fiber (WT), STC2 inhibits PAPP‐A activity suppressing the intracellular signaling of IGF1. Deletion of s*tanniocalcin*‐*2* abrogates this inhibitory mechanism in the *Stc2* ^*−*/*−*^ muscle fibers allowing the PAPP‐A to mobilize IGF1 from the IGF1:IGFBP complex more efficiently. This potentiates the growth promoting IGF1 signaling and stimulates muscle fiber hypertrophy.

Silencing STC2 removes inhibition from the PAPP‐A's proteolytic activity which in turn releases biologically active IGFs from the IGF:IGFBP complex (Jepsen et al., 2015; Kobberø et al., 2022; Oxvig & Conover, 2023) and would promote accrual of muscle mass observed in the *Stc2* ^*−*/*−*^ mice (Figure 4). This increase in muscle mass is consistent with previous studies in humans (Hernandez Cordero et al., 2019), where Hernandez Cordero and colleagues found that a rare variant of the gene was associated with ~0.88 kg increase in appendicular muscle mass. Furthermore, the finding that an *STC2* variant (R44L) has an impaired ability to inhibit PAPP‐A implicates the IGF axis in mediating the effects of STC2 on muscle mass (Marouli et al., 2017). Based on the current model, such STC2 impairment would tend to increase the availability of bioactive IGF1 and IGF2, promoting protein accrual mediated by the PI3K‐Akt/PKB‐mTOR signaling cascade (Rommel et al., 2001). Since the *Stc2* ^*−*/*−*^ have larger muscles compared with wild type mice, it suggests that normally STC2 plays an inhibitory role in the IGF axis to suppress muscle growth. Such a role is consistent with observation that STC2 overexpression in mice reduces muscle mass by 15%–32% (Gagliardi et al., 2005). Moreover, since *Stc2* deletion does not affect the number of fibers, the gene product does not act on the formation of muscle fibers during prenatal development (Ontell et al., 1988; Rowe & Goldspink, 1969). Rather, it affects later processes such as growth and/or maintenance of the muscle fibers once formed, producing the observed increase of fiber CSA. This effect is similar to that of PAPP‐A overexpression (Deb et al., 2012; Rehage et al., 2007), further implicating an inhibitory role of STC2 on the function of PAPP‐A.

Upregulation of endogenous IGF1 mediates hypertrophy of muscle fibers in response to resistance type training in rodents (Adams et al., 1985) and humans (Bamman et al., 2001). Moreover, in a mouse model where several days of overload result in hypertrophy of soleus muscle, the mRNA levels of *IGFBP*‐*4* doubled whereas that of *IGFBP*‐*5* decreased by ~70% (Awede et al., 1999). mRNA of *IGF2* is also increased in human muscles subjected to several weeks of resistance exercise (Zhu et al., 2021). Collectively, these mRNA changes strongly implicate that muscle hypertrophy involves an upregulation of the IGF axis. However, they do not show how these changes in mRNA level translate at the protein level nor whether the level of PAPP‐A and STC2 levels change in the hypertrophy undergoing muscles. The present report gives significant insight into these issues. It shows that the level of the measured proteins of the IGF axis, again with the exception of IGFBP‐5 (which also did not increase in the previous soleus overload study (Awede et al., 1999)), were significantly elevated in the overloaded muscles. An increase in IGF1 and IGF2 was the most prominent effect of overloading, strongly suggesting that this mediated the observed muscle hypertrophy.

The increase in IGF may be a result of increased transcription in the muscle (Awede et al., 1999; Zhu et al., 2021). However, the changes in the profile of other components of the IGF axis suggest that the pericellular delivery of free IGF to its receptor could have contributed to the hypertrophy as well. Specifically, in the overloaded muscles we observed a ~ 48% and ~ 30% increase in the level of IGFBP‐4 and PAPP‐A, respectively. This change would have a synergistic effect in promoting the activation of the IGF axis. PAPP‐A, the only proteinase known to cleave IGF from the IGF:IGFBP‐4 complex, is tethered to cell surface through the glycosaminoglycans (Laursen et al., 2002; Oxvig, 2015). This proximity of PAPP‐A to the cell surface together with an increase in the level of the two proteins, facilitates delivery of the cleaved IGF to its receptor (Laursen et al., 2007). The importance of this pericellular delivery mechanism is illustrated by the individual and double knockout models. The *Papp*‐*a* knockouts are ~35%–40% smaller (Conover et al., 2004; Ning et al., 2008) and knockouts of *Igfbp*‐*4* ~ 17% smaller compared to wild‐type animals, suggesting a growth promoting effect of each of these genes (Ning et al., 2008). However, a double knockout shows an identical growth to the knockouts of *Igfbp*‐*4* (Ning et al., 2008), indicating that IGFBP‐4 contributes to the depletion of free IGF in the absence of PAPP‐A. Therefore, we hypothesize that upregulation of these two proteins would promote muscle hypertrophy through facilitated delivery of free IGF to the receptor.

Another novel and interesting observation was that STC2 levels also increased in the overloaded muscle. We hypothesize that the hypoxia and oxidative stress induced by the activity of the overloaded muscles (Jackson et al., 2016) could have contributed to this increase. It has been shown that hypoxia and oxidative stress upregulate *STC2* transcription in mammalian cells in vitro (Ito et al., 2004). The promotor of *STC2* gene contains the hypoxia response element (Law & Wong, 2010) and the cAMP response element (Ameri & Harris, 2008; Lin & Green, 1988) recognized by transcription factors activated under those conditions, HIF1 and ATF4, respectively (Qie & Sang, 2022). Hence STC2 in the overloaded muscles can play a role of a negative feedback mechanism controlling the PAPP‐A activity and downregulating IGF axis.

The other model used in the study, myostatin dysfunction, showed a similar difference in muscle mass, ~1.8‐fold, as achieved by functional overload, ~1.7‐fold. Despite of this quantitative similarity, the overload model reflects a flux state when the muscles are actively adapting to an overload, whereas in the myostatin deficiency model the BEH strain muscles compared to BEH^*C*/+^ are in a state of maintenance of gradually acquired muscle mass over much longer space of time. It appeared that the molecular mechanism mediating similar muscle mass differences between the two models clearly differed. In the myostatin deficiency model, the levels of IGF1 and IGF2, as well as PAPP‐A were 18%–40% lower in the BEH strain muscles compared with BEH^*C*/+^ mice. Because the BEH strain muscles are larger than BEH^*C*/+^ due to the CSA of muscle fibers (also due to the proportion of the glycolytic fibers but not the total number of fibers) (Lionikas et al., 2013), the IGF axis cannot explain the difference in muscle mass between these two strains. Indeed, the levels of IGF1 and IGF2 in BEH muscle (42 ± 6 pM and 88 ± 14 pM, respectively) were comparable to those in the control muscles of the overload study (39 ± 9 pM and 98 ± 34 pM, respectively) despite of a threefold difference in weight (40.2 mg in BEH vs. 11.9 mg of the control muscles). This apparent paradox is brought about by the role of myostatin. The *Compact* allele in the BEH and BEH^*C*/+^ mice is characterized by a 12‐bp deletion affecting the propeptide sequence which leads to a decrease in mature myostatin (Kocsis et al., 2017). Since myostatin is an inhibitor of the PI3K‐Akt/PKB‐mTOR pathway (Yang et al., 2007), its inhibitory effect on this pathway is substantially reduced in the BEH strain, which is homozygous for the Compact allele, leading to an increase in the Akt/PKB activation (Kocsis et al., 2017). This mechanism might compensate in the BEH mice for the lack of activation via the IGF axis and allow the IGF axis' response had the muscles been subjected to an overload. Indeed, despite the presence of an already significant muscle mass at baseline, the BEH mice respond to overload with muscle hypertrophy (Kilikevicius et al., 2016). Hence, a marked upregulation of the IGF axis appears to be a feature of the muscles undergoing adaptation to overload but not a part of the muscle bulk maintenance programme in BEH mice.

While these results are indicative, our study also has three limitations that should be acknowledged with respect to experimental design and translation into therapeutic potential. First, our analyses were limited by a smaller than ideal sample size. Second, our findings are based on an animal model and some aspects may not be fully transferable to humans. Third, we were not able to characterize the intracellular signaling triggered by the IGF axis in these samples. However, despite these limitations, we have successfully validated a role for *Stc2* in regulating skeletal muscle mass. We have also shown a mechanistic link that explains, at least in part, *STC2*'s association with muscle mass variability observed in humans.

## 5 CONCLUSIONS

Here we show that *Stc2* significantly influences muscle mass via its effect on muscle fiber CSA. We also show that overload‐induced muscle hypertrophy is mediated by the upregulation of both agonists (IGF1, IGF2, IGFBP‐4, PAPP‐A) and antagonists (STC2) of the IGF axis. These data suggest that a suppressive *Stc2* effect on muscle hypertrophy may work as a negative feedback mechanism in resistance exercise.

## AUTHOR CONTRIBUTIONS

Arimantas Lionikas, Claus Oxvig, and Guy S. Bewick conceived the study. Arimantas Lionikas carried out muscle sample analyses. Arimantas Lionikas and Lora K. Heisler generated protein analyses data. Claus Oxvig and Mette Harboe generated and supplied Stc2^−/−^ mouse model. Audrius Kilikevicius conducted synergist ablation study and collected muscle samples. Lutz Bunger developed the BEH and BEL strains and provided the corresponding samples. Arimantas Lionikas, Andrew M. Carroll and Aivaras Ratkevicius collected BEH and BEH^*C*/+^ muscle samples. AIHC carried out statistical analyses. The manuscript was drafted by Arimantas Lionikas with input from all co‐authors.

## CONFLICT OF INTEREST STATEMENT

The authors declare no competing interests.
